# Constrained optimization of divisional load in hierarchically organized tissues during homeostasis

**DOI:** 10.1098/rsif.2021.0784

**Published:** 2022-02-23

**Authors:** Peter Ashcroft, Sebastian Bonhoeffer

**Affiliations:** Institute of Integrative Biology, ETH Zurich, Zurich, Switzerland

**Keywords:** tissue structure, differentiation, somatic mutation, cancer, haematopoiesis, optimization

## Abstract

It has been hypothesized that the structure of tissues and the hierarchy of differentiation from stem cell to terminally differentiated cell play a significant role in reducing the incidence of cancer in that tissue. One specific mechanism by which this risk can be reduced is by minimizing the number of divisions—and hence the mutational risk—that cells accumulate as they divide to maintain tissue homeostasis. Here, we investigate a mathematical model of cell division in a hierarchical tissue, calculating and minimizing the divisional load while constraining parameters such that homeostasis is maintained. We show that the minimal divisional load is achieved by binary division trees with progenitor cells incapable of self-renewal. Contrary to the protection hypothesis, we find that an increased stem cell turnover can lead to lower divisional load. Furthermore, we find that the optimal tissue structure depends on the time horizon of the duration of homeostasis, with faster stem cell division favoured in short-lived organisms and more progenitor compartments favoured in longer-lived organisms.

## 1. Introduction

Many tissues in the human body undergo constant regeneration of their constituent cells. This regeneration allows the tissue to maintain effective function and to prevent damage accumulation, for example from exposure to acidic environments in the gut or ultraviolet light on the skin. In organs such as the colon, skin and haematopoietic system, the regeneration is managed through a hierarchical organization of stem and progenitor cells. These cells undergo amplification of numbers and phenotypic differentiation to produce the functional, terminally differentiated cells (TDC) in large enough quantities to support the functioning of the tissue. The haematopoietic system is the archetypal example of such a tissue: in humans approximately 10^5^ haematopoietic stem cells [1] are ultimately responsible for producing approximately 10^11^ TDC per day to satisfy respiratory, immune and coagulation demands [2–4].

It has been hypothesized that hierarchical population structures have evolved to protect against the accumulation of mutant cells [5–11]. Empirical observations give weight to these claims: despite blood cells being over 10 times more frequent than any other human cell type [12], haematopoietic cancers make up only a small fraction of cancer incidence statistics [13]. It has been suggested that the protection against cancer is conferred by limiting the cumulative number of cell divisions needed to maintain tissue homeostasis [6], as well as ensuring that the majority of cells are transient such that continued cell turnover washes out mutants [5,8].

Mathematical models in which cells are grouped into homogeneous compartments (so-called compartmental models) have been employed to test different aspects of these cancer prevention hypotheses. With such models it is possible to calculate the number of divisions that cells undergo as they differentiate from stem cells to TDC [6,10,14–17]. The mutation risk is inherently linked to this number of divisions; every DNA replication event carries a risk of mistakes, such that more divisions means a higher risk of genetic damage. Once an expression is obtained for the number of cell divisions across the tissue, it is then possible to optimize the tissue architecture in order to minimize the number of divisions and the risk of mutation accumulation [6,14].

Using a computational model, Pepper *et al.* [9] showed that increasing the number of compartments in hierarchically organized tissues limits the somatic evolution (accumulation of multiple beneficial mutations) of those cells. Similarly, Alvarado *et al.* [14] focussed optimizing tissue structures to limit mutation accumulation. They found that the optimal tissue structure depends on the objective of optimization: minimizing the number of cells with a single mutation in the tissue requires a high degree of self-renewal across the compartments, while the time until two-hit mutants are generated is maximized by the non-self-renewing binary division tree. Derényi & Szöllősi [6] were the first to compute the divisional load that is accumulated when producing a lifetime supply of TDC, rather than explicitly focussing on somatic evolution. They found that optimal tissues which minimize divisional load follow a binary tree structure with no propensity for self-renewal among the progenitor cells, as well as a power law increase in differentiation output along the hierarchy.

What is missing is the following: what happens if we constrain the optimization of divisional load by ensuring tissue homeostasis, and what changes if we minimize divisions accumulated after stem cell differentiation or if we consider the total divisions (including stem cells) accumulated across a lifetime? Furthermore, what is the impact of the difference between the timescale on which evolution acts (i.e. until reproductive senescence) and the actual lifetime of an individual on divisional load?

In the next section, we introduce our model of cell proliferation in a hierarchical population structure. For the purpose of our analyses, we primarily consider a reduced model in which only symmetric division events can occur. The full model, which considers symmetric and asymmetric cell divisions, as well as cell death, is described in the electronic supplementary material. We further consider the possibility for stem cells to be non-dividing in the full model, as it has been suggested that quiescence is another mechanism of cancer defence in hierarchical tissues [18]. Using these models, we quantify the number of divisions that cells accumulate. We then use the method of Lagrange multipliers to compute the optimum tissue architecture which minimizes divisional load, while constraining the number of TDC under homeostatic conditions. This optimization is performed on post-stem cell divisions, as well as for total divisions accumulated over a given time horizon.

## 2. Methods

### 2.1. Model structure

**Figure 1.**
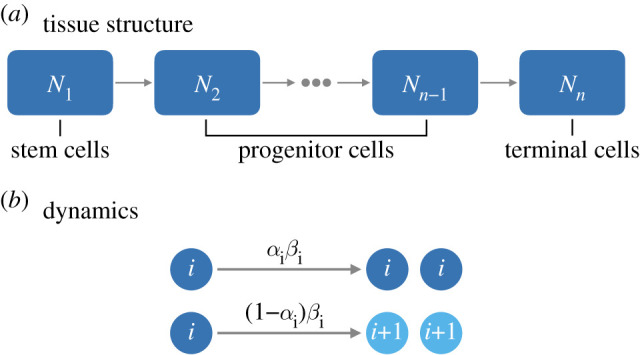
Reduced model schematic. (*a*) We consider a hierarchical population of cells, with stem cells (SC) at the top of the hierarchy giving rise to progenitor cells and ultimately terminally differentiated cells (TDC). (*b*) In this reduced model, cells can only symmetrically self-renew or symmetrically differentiate and there are no quiescent SCs. Here, cells in compartment *i* divide with rate *β*_*i*_. With probability *α*_*i*_ the daughters are both of type *i*, and with complimentary probability 1 − *α*_*i*_ the daughters are of differentiated type *i* + 1. The parameter *β*_*n*_ assumes the role of the death rate of TDCs, where we have *α*_*n*_ = 0. We also ignore SC quiescence in the reduced model.

We consider a compartmental model for cell proliferation in a hierarchical tissue, following the likes of Marciniak-Czochra *et al.* [17], Rodriguez-Brenes *et al.* [19], Stiehl & Marciniak-Czochra [20], Böttcher *et al.* [15], Nienhold *et al.* [21]. Cells are collected into compartments which represent their level of differentiation. Within each compartment, the cells are indistinguishable. The compartments are labelled by *i* ∈ {1, 2, …, *n*}, where *i* = 1 corresponds to stem cells (SC), 1 < *i* < *n* are progenitor cells, and *i* = *n* are terminally differentiated cells (TDC). The number of cells in compartment *i* is denoted as *N*_*i*_. This structure is highlighted in figure 1*a*. We note that we only consider a linear population structure, with population branching here ignored to reduce the number of model parameters. In the full model, we additionally consider a compartment of non-dividing (quiescent) SCs, labelled *i* = 0. See appendix I in the electronic supplementary material for details.

### 2.2. Dynamics

Cells move between compartments through differentiation, which is strictly coupled to cell division in this model. A division event produces two daughter cells, each of which may remain in the same compartment as the mother cell (*i*), or differentiate to the next compartment along the hierarchy (*i* + 1). We do not consider the possibility of de-differentiation (i.e. cells cannot move from compartment *i* to compartment *i* − 1). De-differentiation would lead to an increase in divisions accumulated during homeostasis compared to a forward-only differentiating tissue.

To build intuition and guide analytical calculations, we primarily consider the reduced model (figure 1*b*) in which cells only divide symmetrically. We ignore cell death and asymmetric cell division, as well as SC quiescence compared to the full model that is described in appendix I in the electronic supplementary material. This reduced model is parametrized by *β*_*i*_, which is the division rate of cells in compartment *i*, and *α*_*i*_, which is their probability of self-renewal (correspondingly, 1 − *α*_*i*_ is the probability of differentiation) following a division event. The reduced model is described by the set of ordinary differential equations

2.1 $${\dot{N}}_{i}=2(1-\alpha_{i-1})\beta_{i-1}N_{i-1}-(1-2\alpha_{i})\beta_{i}N_{i},$$

where the first term describes the inflow of two daughter cells from compartment *i* − 1 and the second term is the net loss rate (differentiation minus self-renewal) of cells from compartment *i*. Furthermore, we set *α*_*n*_ = 0 and the parameter *β*_*n*_ assumes the role of the death rate of the TDCs.

Under homeostasis the number of cells per compartment does not change (${\dot{N}}_{i}=0$). The steady state of equation (2.1), which we write as $N_{i}^{*}$, satisfies the relation

2.2 $$(1-2\alpha_{i})\beta_{i}N_{i}^{*}=2(1-\alpha_{i-1})\beta_{i-1}N_{i-1}^{*},$$

i.e. the net loss from compartment *i* is balanced by the influx from differentiating cells in compartment *i* − 1. For this steady state to exist, we require *α*_1_ = 0.5 and *α*_*i*_ < 0.5 for 1 < *i* < *n*. Solving equation (2.2) recursively, we have

2.3 $$\begin{array}{rl} N_{i}^{*} & =\frac{\beta_{1}}{\beta_{i}}\left[\prod_{k=1}^{i-1}\frac{2(1-\alpha_{k})}{1-2\alpha_{k+1}}\right]N_{1}^{*}\\ & =\frac{1}{1-2\alpha_{i}}\frac{\beta_{1}}{\beta_{i}}\left[\prod_{k=2}^{i-1}\frac{2(1-\alpha_{k})}{1-2\alpha_{k}}\right]N_{1}^{*}. \end{array}$$

In particular, for TDCs (*i* = *n*), we have

2.4 $$N_{n}^{*}=\frac{\beta_{1}}{\beta_{n}}\left[\prod_{k=2}^{n-1}\frac{2(1-\alpha_{k})}{1-2\alpha_{k}}\right]N_{1}^{*}.$$

### 2.3. Counting divisions

For compartmental models, the expected number of divisions that cells accumulate in a tissue has previously been calculated using two methods: firstly, Derényi & Szöllősi [6] constructed ordinary differential equations (ODE) for the cumulative number of divisions across all cells in a compartment, and from this they extracted the average number of divisions that cells undergo during their lifetime. Alternatively, Böttcher *et al.* [15] constructed an explicit set of ODEs for sub-populations of cells of type *i* that have undergone *j* division events, from which the mean number of divisions per cell is easily extracted. These models yield identical results for the mean number of divisions in the reduced and full models, as described in appendices II and III in the electronic supplementary material, respectively.

Briefly, following the method of Derényi & Szöllősi [6], the cumulative number of divisions accumulated across all SCs, *T*_1_, in the reduced model satisfies the ODE

2.5 $${\dot{T}}_{1}=\alpha_{1}\beta_{1}N_{1}^{*}\left[\frac{T_{1}}{N_{1}^{*}}+2\right]-(1-\alpha_{1})\beta_{1}N_{1}^{*}\left[\frac{T_{1}}{N_{1}^{*}}\right]=\beta_{1}N_{1}^{*},$$

where we have used *α*_1_ = 0.5. Together with the initial condition *T*_1_(0) = 0, equation (2.5) can be solved to give $T_{1}(t)=\beta_{1}N_{1}^{*}t$. Hence, the average number of divisions per SC at time *t*, *D*_1_(*t*), satisfies

2.6 $$D_{1}(t)=\frac{T_{1}(t)}{N_{1}^{*}}=\beta_{1}t,$$

i.e. SC divisions are accumulated linearly with time.

The derivation of the equations for ${\dot{T}}_{i}$ [similar to equation (2.5)] for all compartments 1 ≤ *i* ≤ *n* is described in appendix II in the electronic supplementary material. Ultimately, the expected number of divisions that cells accumulate after a time period *t*, *D*_*i*_(*t*), is described by a set of linear ODEs which can be solved analytically. However, the resulting expression becomes increasingly complicated for large *n*. Instead, we here make the assumption that the SC dynamics are much slower than non-SCs. We can then express the mean number of divisions per TDC at time *t*, *D*_*n*_(*t*), as the sum of the SC divisions up to that point in time (equation (2.6)) plus the expected number of divisions that are accumulated *after* SC differentiation, *D*_*n*_. The latter term can be calculated (see appendix II in the electronic supplementary material) as

2.7 $$D_{n}=\sum_{\;j=2}^{n}\frac{1}{1-2\alpha_{\;j}}.$$

Hence, in the reduced model, the divisions accumulated by TDCs after time *t* is approximated by

2.8 $$D_{n}(t)\approx D_{\mathrm{SC}}(t)+D_{n}=\beta_{1}t+\sum_{\;j=2}^{n}\frac{1}{1-2\alpha_{\;j}}.$$

This equation is equivalent to the result of Derényi & Szöllősi [6] for the reduced dynamics. The calculation differs from Derényi & Szöllősi [6] in the optimization procedure in the next steps.

### 2.4. Optimizing tissue architecture

Given the expressions for the expected number of divisions accumulated per TDC, we can minimize these values—subject to physical constraints—to deduce the optimal tissue structure parameters which minimize the divisional load. The constraint that we apply is that during homoeostasis the tissue should maintain a given number of TDCs, such that the tissue remains functional. For the reduced model, we derive the constraint from the equilibrium TDC population size, equation (2.4), which we rearrange as

2.9 $$\prod_{k=2}^{n-1}\frac{2(1-\alpha_{k})}{1-2\alpha_{k}}-\frac{\beta_{n}N_{n}^{*}}{\beta_{1}N_{1}^{*}}=0.$$

The parameters *α*_*i*_ (1 < *i* < *n*), *β*_1_, $N_{1}^{*}$ and *n* must satisfy this relation to ensure that enough TDCs are present in the tissue. We keep the number of TDCs ($N_{n}^{*}$) and the death rate of TDCs (*β*_*n*_) fixed, as well as setting *α*_1_ = 0.5 and *α*_*n*_ = 0 as required in the model definition.

We then use the method of Lagrange multipliers [22] to constrain the homeostatic number of TDCs and minimize the tissue’s divisional load. The objective function that is to be optimized is the number of divisions accumulated per TDC, which we can construct in one of two ways: firstly, we can consider only the divisions accumulated after a SC has differentiated, *D*_*n*_ (equation (2.7)). In this scenario, we drop the time dependence and only focus on optimizing the differentiation structure of the tissue. The Lagrangian function is then constructed as the linear combination of equations (2.7) and (2.9), i.e.

2.10 $$L=\sum_{\;j=2}^{n}\frac{1}{1-2\alpha_{\;j}}-\lambda \left[\prod_{k=2}^{n-1}\frac{2(1-\alpha_{k})}{1-2\alpha_{k}}-\frac{\beta_{n}N_{n}^{*}}{\beta_{1}N_{1}^{*}}\right],$$

where the coefficient *λ* is the Lagrange multiplier.

Secondly, we can include SC divisional history and ask how many divisions have TDCs accumulated after a given time *t*, *D*_*n*_(*t*), using the approximation in equation (2.8) as the objective function. The Lagrangian function in this case is

2.11 $$L(t)=\beta_{1}t+\sum_{\;j=2}^{n}\frac{1}{1-2\alpha_{\;j}}-\lambda \left[\prod_{k=2}^{n-1}\frac{2(1-\alpha_{k})}{1-2\alpha_{k}}-\frac{\beta_{n}N_{n}^{*}}{\beta_{1}N_{1}^{*}}\right].$$

The optimum tissue architecture is then found by computing the stationary points of L or L(t) considered as a function of *α*_*i*_ (1 < *i* < *n*), *β*_1_, and the Lagrange multiplier *λ*. Due to the discrete nature of *n*, we cannot identify its optimum value through stationary point analysis. We can, however, identify it through graphical methods. The number of SCs does not appear in the objective function in the Lagrangian, so this too must be optimized by graphical methods.

The Lagrangian functions for the full model can be constructed analogously. See appendix V in the electronic supplementary material for details.

## 3. Results

Although our results are algebraic, we illustrate these results using the observed parameters of human erythropoiesis (red blood cell production). A healthy human has approximately 10^13^ red blood cells at any given time [12] and these cells have a lifetime of approximately 120 days [3]. The most recent estimates of human haematopoietic stem cell (HSC) numbers are approximately 10^5^ cells which divide approximately once per year [1].

### 3.1. Optimized tissue architectures in the reduced model

#### 3.1.1. Additional divisions after stem cell differentiation

To determine the architecture that minimizes the divisional load, one needs to find the stationary points of the Lagrangian function in equation (2.10). These stationary points satisfy $\partial L/\partial \alpha_{i}=0$ (1 < *i* < *n*) and ∂L/∂λ=0. As the SC division rate *β*_1_ features only in the constraint and not in the objective function, this parameter cannot be optimized through stationary point analysis. The values of the *α*_*i*_ at the stationary point of L are the parameters which minimize the number of divisions accumulated by the TDCs after leaving the SC compartment, while still maintaining the homeostatic population of TDCs.

From the $\partial L/\partial \alpha_{i}=0$ (for 1 < *i* < *n*) condition, we arrive at

3.1 $$\frac{2(1-\alpha_{i})}{1-2\alpha_{i}}=\lambda \prod_{k=2}^{n-1}\frac{2(1-\alpha_{k})}{1-2\alpha_{k}},$$

which is only satisfied if

3.2 $$\frac{2(1-\alpha_{i})}{1-2\alpha_{i}}=\lambda^{1/(3-n)}.$$

This implies that all *α*_*i*_ are equal for 1 < *i* < *n* in a tissue which minimizes the divisional load after SC differentiation. This equivalence of the *α*_*i*_ across compartments was used by Böttcher *et al.* [15] and Alvarado *et al.* [14] as a simplifying assumption without derivation. Furthermore, this result is consistent with the result of Derényi & Szöllősi [6], who found that the ratio of differentiation output between two consecutive compartments is constant in optimized tissues.

The value of *λ*, and ultimately the value of the *α*_*i*_, are obtained from the ∂L/∂λ=0 condition, which is equal to the population size constraint (equation (2.9)). By substituting equation (3.2) into equation (2.9) and then solving for *λ*, we find the value of the Lagrange multiplier

3.3 $$\lambda ={\left(\frac{\beta_{n}N_{n}^{*}}{\beta_{1}N_{1}^{*}}\right)}^{\left(3-n)/(n-2\right)}.$$

By rearranging equation (3.2), we can now define the values of *α*_*i*_ which minimize the number of divisions per TDC during homeostasis and for a given number of compartments. These values will depend explicitly on the number of compartment size *n*, hence we write this value as $\hat{\alpha }(n)$, which satisfies

3.4 $$\hat{\alpha }(n)=\frac{\lambda^{1/(3-n)}-2}{2\lambda^{1/(3-n)}-2}=\frac{{\left(\frac{\beta_{n}N_{n}^{*}}{\beta_{1}N_{1}^{*}}\right)}^{1/(n-2)}-2}{2{\left(\frac{\beta_{n}N_{n}^{*}}{\beta_{1}N_{1}^{*}}\right)}^{1/(n-2)}-2}.$$

We here want to differentiate between the subscript notation, which is just the index of the compartment, and the bracket notation, denoting that the optimized parameter value $\hat{\alpha }(n)$ depends on the number of compartments in the hierarchy.

From equation (3.4), we see that an increase in the number of compartments *n* must be compensated by a decrease in the progenitor self-renewal probability $\hat{\alpha }(n)$ to maintain the homeostatic level of TDCs. However, $\hat{\alpha }(n)$ is a probability and is bounded from below by zero. Once *n* is increased sufficiently such that ${\left(\beta_{n}N_{n}^{*}/\beta_{1}N_{1}^{*}\right)}^{\frac{1}{n-2}}<2$, we have to artificially set $\hat{\alpha }(n)=0$ and there will be a new (increased) steady state TDC population size ${\hat{N}}_{n}$. From equation (2.4), the number of TDCs will satisfy

3.5 $${\hat{N}}_{n}=\frac{\beta_{1}N_{1}^{*}}{\beta_{n}}{\left(\frac{2[1-\hat{\alpha }(n)]}{1-2\hat{\alpha }(n)}\right)}^{n-2},$$

while the number of divisions per TDC after SC differentiation (equation (2.7)) satisfies

3.6 $${\hat{D}}_{n}=(n-2)\frac{1}{1-2\hat{\alpha }(n)}+1.$$

The minimum number of divisions occurs when $\hat{\alpha }(n)$ first reaches zero, i.e. when ${\left(\beta_{n}N_{n}^{*}/\beta_{1}N_{1}^{*}\right)}^{1/(n-2)}=2$. At this point, the number of compartments is given by

3.7 $$\hat{n}=\log_{2}\left(\frac{\beta_{n}N_{n}^{*}}{\beta_{1}N_{1}^{*}}\right)+2,$$

and the divisional load per TDC is

3.8 $${\hat{D}}_{\hat{n}}=\log_{2}\left(\frac{\beta_{n}N_{n}^{*}}{\beta_{1}N_{1}^{*}}\right)+1=\hat{n}-1.$$

Therefore, perhaps unsurprisingly, the binary division tree ($\hat{\alpha }(n)=0$) with minimal number of compartments ($n=\hat{n}$) is the tissue structure which minimizes divisional load while maintaining the TDC population. As shown by Böttcher *et al.* [15], both the mean and variance of the cumulative number of cell divisions is minimal for this non-self-renewing structure. If the number of compartments *n* is smaller than $\hat{n}$, then there must be some self-renewal in the progenitor compartments, which leads to an increase in the cumulative number of divisions. If $n>\hat{n}$, then there are more divisions than required to maintain the TDC population and the tissue produces more TDCs than necessary.

**Figure 2.**
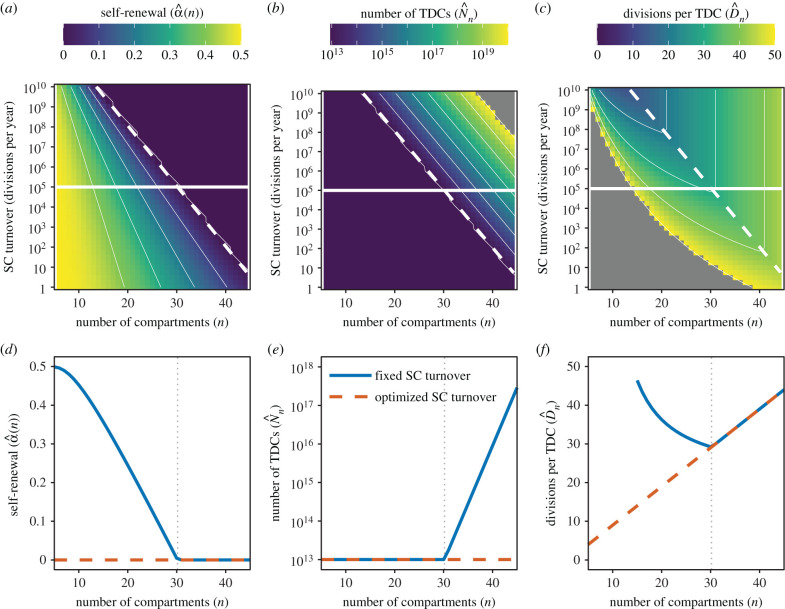
(*a*) Self-renewal probability ($\hat{\alpha }(n)$ ), (*b*) TDC population size (${\hat{N}}_{n}$) and (*c*) cumulative number of divisions per TDC after SC differentiation (${\hat{D}}_{n}$ ) as a function of the number of compartments *n* and SC turnover (total divisions per year) $\beta_{1}N_{1}^{*}$ for the reduced model. Here, we have fixed the TDC properties $\beta_{n}=1/120\;{\mathrm{day}}^{-1}$ and $N_{n}^{*}=10^{13}\;\mathrm{cells}$, which are approximate numbers for red blood cells in humans [3]. The horizontal line is the approximate SC turnover in humans ($N_{1}^{*}=10^{5}\;\mathrm{cells}$; *β*_1_ = 1 yr^−1^; Lee-Six *et al.* [1]). The diagonal dashed line represents the optimum number of compartments $\hat{n}$ for a given SC turnover which minimizes the cumulative number of divisions (equation (3.7)). The values of $\hat{\alpha }(n)$, ${\hat{N}}_{n}$ and ${\hat{D}}_{n}$ along these solid and dashed lines are shown in (*d*–*f*), respectively. Colour scales for ${\hat{N}}_{n}$ and ${\hat{D}}_{n}$ are truncated for visual clarity.

The values of $\hat{\alpha }(n)$ (probability of self-renewal that minimizes divisional load per TDC), ${\hat{N}}_{n}$ (the corresponding number of TDCs) and ${\hat{D}}_{n}$ (the divisional load per TDC) are illustrated in figure 2*a*–*c* as a function of total SC turnover ($\beta_{1}N_{1}^{*}$) and the number of compartments in the tissue (*n*). The line described by equation (3.7) (dashed diagonal) does indeed follow the minimum divisional load; any change in *either* SC turnover or the number of compartments leads to an increase in divisional load. However, by moving along this line (equation (3.7)), in particular to higher SC turnover and lower number of compartments, the divisional load can be reduced, as shown in figure 2*f*. Therefore, the tissues which minimize divisional load after SC differentiation would have very high SC turnover and few differentiation steps.

If the SC turnover is fixed for another reason (e.g. due to spatial and nutritional niche competition), then the minimum divisional load occurs when there are enough compartments such that there is no progenitor self-renewal ($\hat{\alpha }(n)=0$), but not too many such that TDCs are not overproduced (${\hat{N}}_{n}=N_{n}^{*}$) (figure 2*d*–*f*).

#### 3.1.2. Cumulative divisions after lifetime *t*

The objective function in the Lagrangian L(t) (equation (2.11)), which now counts SC divisions too, is explicitly dependent on the SC division rate parameter *β*_1_, as well as on the organism’s lifetime. We can now determine the parameters and tissue structure which minimize the divisional load after a time period *τ*, which we call the timescale of optimization. The optimum parameter values must satisfy $\partial L(\tau )/\partial \alpha_{i}=0$ (1 < *i* < *n*), ∂L(τ)/∂λ=0, as well as $\partial L(\tau )/\partial \beta_{1}=0$. Note that *τ* is the timescale of optimization: it is entirely possible that the organism lives for a shorter (*t* < *τ*) or longer (*t* > *τ*) time and we therefore have to consider two timescales.

The partial derivative of L(τ) (equation (2.11)) with respect to *α*_*i*_ are unchanged from the previous section, such that the condition equation (3.2) must be satisfied. From the condition $\partial L(\tau )/\partial \beta_{1}=0$, we arrive at

3.9 $$\beta_{1}=\sqrt{\frac{\lambda }{\tau }\frac{\beta_{n}N_{n}^{*}}{N_{1}^{*}}}.$$

Note that the SC division rate *β*_1_ is now decoupled from the SC population size $N_{1}^{*}$ for this time-dependent problem, and therefore we can no longer consider SC turnover $\beta_{1}N_{1}^{*}$ as an independently variable quantity as was done in figure 2.

These values of *α*_*i*_ (equation (3.2)) and *β*_1_ (equation (3.9)) can be substituted into the population size constraint equation (2.9), i.e. the ∂L(τ)/∂λ=0 condition, and we can solve for the value of the Lagrange multiplier *λ*:

3.10 $$\lambda ={\left(\tau \frac{\beta_{n}N_{n}^{*}}{N_{1}^{*}}\right)}^{\left(3-n)/(n-1\right)}.$$

The values of *α*_*i*_ and *β*_1_ which minimize the number of divisions per TDC after time *τ* are thus given by

3.11 $$\begin{array}{rl} & \alpha_{i}=\hat{\alpha }(n)=\frac{{\left(\tau \beta_{n}N_{n}^{*}/N_{1}^{*}\right)}^{1/(n-1)}-2}{2{\left(\tau \beta_{n}N_{n}^{*}/N_{1}^{*}\right)}^{1/(n-1)}-2}\;\mathrm{and}\\ & {\hat{\beta }}_{1}(n)=\frac{1}{\tau }{\left(\tau \frac{\beta_{n}N_{n}^{*}}{N_{1}^{*}}\right)}^{1/(n-1)}. \end{array}$$

To satisfy $\hat{\alpha }(n)\geq 0$, we require ${\left(\tau \beta_{n}N_{n}^{*}/N_{1}^{*}\right)}^{1/(n-1)}\geq 2$. This condition imposes ${\hat{\beta }}_{1}(n)\geq 2/\tau$. Again, we here want to clarify the difference between the subscript notation, which is just the index of the compartment, and the bracket notation, denoting that the optimized parameter values $\hat{\alpha }(n)$ and ${\hat{\beta }}_{1}(n)$ depend on the number of compartments in the hierarchy.

From equation (2.4), the number of TDCs will satisfy

3.12 $${\hat{N}}_{n}=\frac{{\hat{\beta }}_{1}(n)N_{1}^{*}}{\beta_{n}}{\left(\frac{2[1-\hat{\alpha }(n)]}{1-2\hat{\alpha }(n)}\right)}^{n-2},$$

while the number of divisions per TDC after time *t* (equation (2.8)) satisfies

3.13 $${\hat{D}}_{n}(t)={\hat{\beta }}_{1}(n)t+(n-2)\frac{1}{1-2\hat{\alpha }(n)}+1.$$

The minimum number of divisions occurs when $\hat{\alpha }(n)=0$ and ${\hat{\beta }}_{1}(n)=2/\tau$, or when ${\left(\tau \beta_{n}N_{n}^{*}/N_{1}^{*}\right)}^{\frac{1}{n-1}}=2$. At this point, the number of compartments is given by

3.14 $$\hat{n}=\log_{2}\left(\frac{\tau \beta_{n}N_{n}^{*}}{N_{1}^{*}}\right)+1,$$

and the divisional load per TDC is

3.15 $${\hat{D}}_{\hat{n}}(t)=2\frac{t}{\tau }+\hat{n}-1.$$

**Figure 3.**
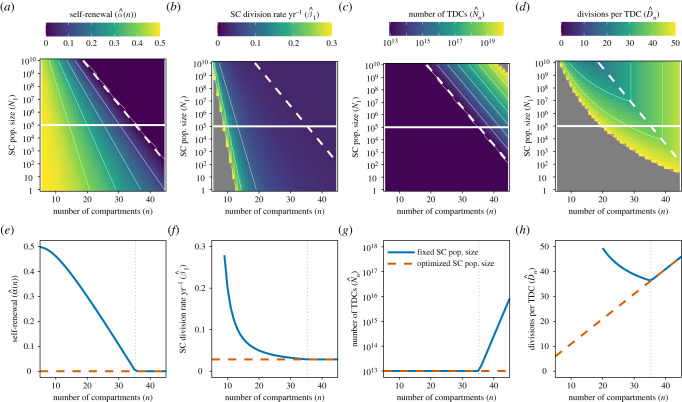
(*a*) Self-renewal probability ($\hat{\alpha }(n)$), (*b*) SC division rate (${\hat{\beta }}_{1}$), (*c*) TDC population size (${\hat{N}}_{n}$ ) and (*d*) cumulative number of divisions per TDC after time t=τ=70 years (${\hat{D}}_{n}(t)$) as a function of the number of compartments *n* and SC population size $N_{1}^{*}$ for the reduced model. These parameters/variables are described by equations (3.11)–(3.13). Here, we have fixed the TDC properties $\beta_{n}=1/120\;{\mathrm{day}}^{-1}$ and $N_{n}^{*}=10^{13}\;\mathrm{cells}$, which are approximate numbers for red blood cells in humans [3]. The horizontal line in (*a*–*d*) is the approximate SC population size in humans ($N_{1}^{*}=10^{5}\;\mathrm{cells}$; Lee-Six *et al.* [1]). The diagonal dashed line represents the optimum number of compartments $\hat{n}$ for a given SC population size which minimizes the cumulative number of divisions (equation (3.14)). The values of $\hat{\alpha }(n)$, ${\hat{\beta }}_{1}(n)$, ${\hat{N}}_{n}$ and ${\hat{D}}_{n}(t)$ along these solid and dashed lines are shown in (*e*–*h*), respectively. Colour scales for ${\hat{\beta }}_{1}(n)$, ${\hat{N}}_{n}$, ${\hat{D}}_{n}(t)$ are truncated for visual clarity.

Firstly, we consider the optimized parameters and divisional load when the lifetime is equal to the optimization timescale (*t* = *τ* = 70 years). For this case, the values of $\hat{\alpha }(n)$, ${\hat{\beta }}_{1}(n)$, ${\hat{N}}_{n}$ and ${\hat{D}}_{n}$ are illustrated in figure 3*a*–*d* as a function of SC number ($N_{1}^{*}$) and the number of compartments in the tissue (*n*). The line described by equation (3.14) (dashed diagonal) again follows the minimum divisional load; any change in *either* SC population size or the number of compartments leads to an increase in divisional load. However, by moving along this line (equation (3.14), in particular to higher SC population size and lower number of compartments, the divisional load per TDC can be reduced, as shown in figure 3*h*. Therefore, the tissues which minimize TDC divisional load have many SCs and few differentiation steps. The optimum SC division rate ${\hat{\beta }}_{1}(n)$ is substantially slower than the observed once per year [1], with SCs dividing twice per lifetime in the optimized tissue.

For a fixed SC population size, the SC division rate is a decreasing function of the number of compartments, such that shorter hierarchies require more input from the SC compartment (figure 3*f*). The minimum divisional load occurs when there are enough compartments such that there is no progenitor self-renewal ($\hat{\alpha }(n)=0$), but not too many such that TDCs are not overproduced (${\hat{N}}_{n}=N_{n}^{*}$), and when SCs are dividing as slowly as possible (figure 3*e*–*h*).

**Figure 4.**
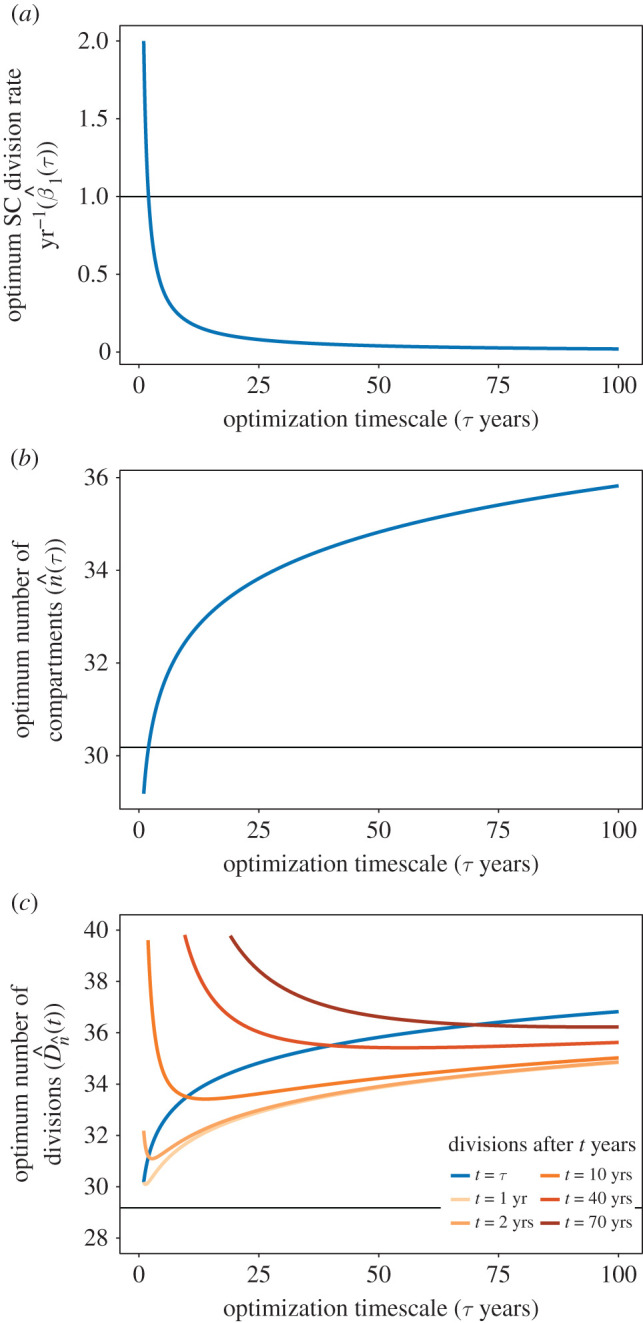
(*a*) Optimum SC division rate (${\hat{\beta }}_{1}$), (*b*) number of compartments ($\hat{n}$ ) and (*c*) TDC divisional load (${\hat{D}}_{\hat{n}}(t)$) as functions of the optimization timescale *τ*. The optimum self-renewal probability $\hat{\alpha }(n)=0$ is not shown. Here, we have fixed the TDC properties $\beta_{n}=1/120\;{\mathrm{day}}^{-1}$ and $N_{n}^{*}=10^{13}\;\mathrm{cells}$, which are approximate numbers for red blood cells in humans [3], as well as the SC population size $N_{1}^{*}=10^{5}\;\mathrm{cells}$ [1]). The horizontal line in (*a*) represents the estimated SC division rate in humans ($\beta_{1}=1\;{\mathrm{yr}}^{-1}$; Lee-Six *et al.* [1]). Horizontal lines in (*b*,*c*) represent the number of compartments and the divisional load when we optimize for divisions accumulated after SC differentiation (i.e. without time).

The optimum progenitor self-renewal rate is $\hat{\alpha }(\hat{n})=0$. However, the optimum SC division rate ${\hat{\beta }}_{1}(\hat{n})$, as well as the optimum number of compartments in the hierarchy, $\hat{n}$, depend on the timescale of optimization *τ*. As this timescale increases, the optimum SC activity decreases (${\hat{\beta }}_{1}(\hat{n})=2/\tau$; figure 4*a*) and the number of amplification steps required to maintain homeostasis increases ($\hat{n}=\log_{2}(\tau \beta_{n}N_{n}^{*}/N_{1}^{*})+1$;figure 4*b*). These properties are independent of the lifetime of the individual. However, the lifetime *t* does contribute to the accumulated divisional load (equation (3.15)). When we fix *t* = *τ*, we have ${\hat{D}}_{\hat{n}}(\tau )=\hat{n}(\tau )+1$, which increases logarithmically with *τ* (blue line in figure 4*c*). If the lifetime *t* exceeds the optimization timescale *τ* (for example when an individual lives beyond reproductive senescence), then TDCs accumulate additional divisions at a rate of 2/*τ* per year (the SC division rate). For short optimization timescales *τ*, this means a rapid accumulation of additional divisions (orange lines in figure 4*c*).

**Figure 5.**
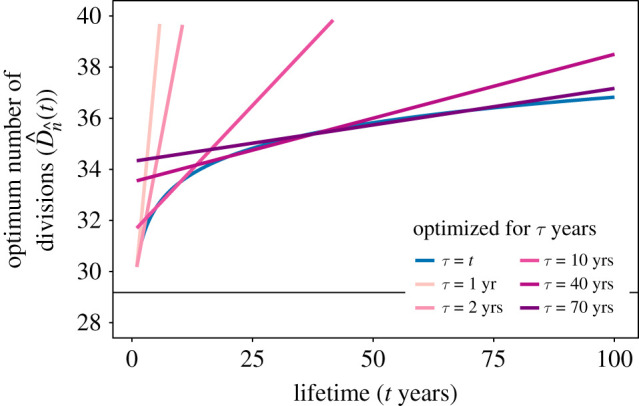
Optimum TDC divisional load (${\hat{D}}_{\hat{n}}(t)$) as a function of lifetime *t* for various optimization timescales *τ* (colours). Here, we have fixed the TDC properties $\beta_{n}=1/120\;{\mathrm{day}}^{-1}$ and $N_{n}^{*}=10^{13}\;\mathrm{cells}$, which are approximate numbers for red blood cells in humans [3], as well as the SC population size $N_{1}^{*}=10^{5}\;\mathrm{cells}$ [1]). The horizontal line represented the TDC divisional load when we optimize for divisions accumulated after SC differentiation (i.e. without time).

This rapid accumulation of divisions can be seen more clearly by plotting ${\hat{D}}_{\hat{n}}(t)$ as a function of lifetime *t* for different optimization timescales *τ* (figure 5). We also see that choosing an optimization timescale *τ* that exceeds the lifetime *t* results in more accumulated divisions than necessary in early life, due to the increased number of differentiation steps.

### 3.2. Optimized architectures in the full model

For the full model (see appendix I in the electronic supplementary material for parameter descriptions), including quiescence and asymmetric division, we can repeat the above analyses using the Lagrangian function

3.16 $$\begin{array}{rl} L & =\sum_{\;j=2}^{n-1}\left(\frac{2b_{\;j}+c_{\;j}}{b_{\;j}-a_{\;j}}-1\right)+1\\ & \;-\lambda \left[\left(\prod_{k=2}^{n-1}\frac{2b_{k}+c_{k}}{b_{k}-a_{k}}\right)-\frac{d_{n}N_{n}^{*}}{(2b_{1}+c_{1})(\gamma /(\ell +\gamma ))N_{\mathrm{SC}}^{*}}\right], \end{array}$$

for optimizing the divisional load after SC differentiation, and

3.17 $$L(t)=(2b_{1}+c_{1})\frac{\gamma }{\ell +\gamma }t+L,$$

for optimizing divisional load after a lifetime *t*. Note that cell death would have to be compensated by additional cell divisions and will result in an increase in the average number of divisions of the surviving cells. We have therefore immediately set the death rate *d*_*i*_ = 0 for 1 ≤ *i* < *n*. We have also used the condition that SC self-renewal and SC differentiation occur at the same rate, *a*_1_ = *b*_1_, which is required for homeostasis.

The optimization process is described in full in appendix V in the electronic supplementary material. From this analysis, we find that the ratio

3.18 $$\phi_{i}=\frac{2b_{i}+c_{i}}{b_{i}-a_{i}},$$

is conserved across all progenitor compartments in optimized tissues, i.e. $\phi_{i}=\hat{\phi }(n)$ for 1 < *i* < *n*. Because of the conditions *b*_*i*_ − *a*_*i*_ > 0, as well as *a*_*i*_ ≥ 0, *b*_*i*_ ≥ 0, and *c*_*i*_ ≥ 0, this ratio is bounded by $2\leq \hat{\phi }(n)<\infty$. When considering only divisions accumulated after SC differentiation (i.e. without time dependence), this ratio takes the value

3.19 $$\hat{\phi }(n)={\left(\frac{d_{n}N_{n}^{*}}{(2b_{1}+c_{1})(\gamma /(\ell +\gamma ))N_{\mathrm{SC}}^{*}}\right)}^{1/(n-2)},$$

and the divisional load per TDC is

3.20 $${\hat{D}}_{n}=(n-2)[\hat{\phi }(n)-1]+1.$$

This divisional load is minimized when $\hat{\phi }(n)=2$, which can only be achieved if *a*_*i*_ = *c*_*i*_ = 0, i.e. progenitor cells (1 < *i* < *n*) do not self-renew or divide asymmetrically, they only differentiate. This pattern was also described by Derényi & Szöllősi [6]. By increasing the SC differentiation flux $(2b_{1}+c_{1})(\gamma /(\ell +\gamma ))N_{\mathrm{SC}}^{*}$, we can reduce the number of compartments while maintaining the TDC population, hence reducing the divisional load (as described for the reduced model).

When we optimize the tissue architecture based on the total TDC divisional load including SC divisions, we have to include an optimization timescale *τ*. From our analysis (appendix V in the electronic supplementary material), we find the following relations for the ratio $\hat{\phi }(n)$ and the total SC division rate

3.21 $$\begin{array}{rl} & \hat{\phi }(n)={\left(\tau \frac{d_{n}N_{n}^{*}}{N_{\mathrm{SC}}^{*}}\right)}^{1/(n-1)}\;\mathrm{and}\\ & \;(2b_{1}+c_{1})\frac{\gamma }{\ell +\gamma }=\frac{1}{\tau }{\left(\tau \frac{d_{n}N_{n}^{*}}{N_{\mathrm{SC}}^{*}}\right)}^{1/(n-1)}, \end{array}$$

and the divisional load after a lifetime *t* satisfies

3.22 $${\hat{D}}_{n}(t)=(2b_{1}+c_{1})\frac{\gamma }{\ell +\gamma }t+(n-2)[\hat{\phi }(n)-1]+1.$$

From the relation for the total SC division rate, we observe that there is a trade-off between the division rate of active SCs (2*b*_1_ + *c*_1_) and the fraction of actively dividing SCs [*γ*/(ℓ + *γ*)]: if active SCs are dividing faster, then the number of actively dividing cells can be smaller. The total SC division rate is bounded by 2/*τ* ≤ (2*b*_1_ + *c*_1_)(*γ*/(ℓ + *γ*)) < ∞. The TDC divisional load is minimized when $\hat{\phi }(n)=2$, (2*b*_1_ + *c*_1_)(*γ*/(ℓ + *γ*)) = 2/*τ* and $\hat{n}=\log_{2}(\tau d_{n}N_{n}^{*}/N_{\mathrm{SC}}^{*})+1$.

## 4. Discussion

In this study, we set out to quantify and then minimize the divisional load that is accumulated in TDC in a homeostatic tissue. For insight, we focussed on a reduced dynamical model in which a division event either lead to symmetric self-renewal or symmetric differentiation. Homeostasis was enforced by constraining the number of progenitor cell compartments (i.e. differentiation steps), self-renewal probabilities per compartment and division rates per compartment such that the TDC population remains at its steady-state level.

In tissues that minimize the divisional load, we find that the self-renewal probability is constant throughout all progenitor cell compartments. The optimum number of compartments is then the smallest number for which progenitor self-renewal can be zero without producing excess TDCs. Therefore, the optimum tissue structure that minimizes divisional load is a binary division tree. By increasing SC turnover, either by increasing SC numbers or SC division rate, the number of progenitor compartments that are required to amplify cell numbers can be reduced and subsequently the divisional load of TDCs is also reduced. Therefore, if optimizing purely based on divisional load as we have done here, it would make sense to have a high-turnover SC compartment, which is the opposite of what we actually observe in, e.g. the haematopoietic system or colonic crypts. Hence, there must be other selection pressures or physiological constraints at play that result in the observed low SC turnover in human tissues.

The above results were obtained based on the divisional load accumulated in the tissue after SC differentiation and in the absence of a time component. However, in our model, SCs accumulate divisions linearly with time. When including these SC divisions, we find that in the short term it is better to have higher SC turnover and fewer progenitor compartments, but in the long term having more compartments and less SC turnover is optimal. Therefore, based on our optimization criteria, we would expect that in longer-lived organisms (with the same homeostatic TDC number) SCs should divide more slowly and there should be a greater number of amplification steps to minimize division accumulation. A recent measurement of somatic mutation rate across animals with different lifespans supports this hypothesis, with longer-lived animals having a lower rate of somatic mutation accumulation [23]. Furthermore, we find that underestimating the timescale of optimization relative to lifetime leads to a large increase in divisional load at ages beyond the optimization timescale.

Our full model of cell dynamics also allows asymmetric division, cell death and SC quiescence. Repeating the above analysis, we find that the ratio of differentiation output to net loss in a progenitor compartment is conserved across progenitor compartments in optimized tissues. This is a more general principle than derived by Derényi & Szöllősi [6] who found that the ratio of differentiation output between two consecutive progenitor compartments is constant in optimized tissues. Furthermore, the minimum divisional load is achieved when the differentiation output to net loss ratio is equal to two, i.e. when progenitor cells do not self-renew and differentiate only symmetrically. This is something that could be compared to empirical data to check for tissue optimization, if such data existed. Finally, from our model and analysis, we find that there is a trade-off between the SC division rate and the fraction of quiescent SCs: faster dividing SCs would require a larger quiescent fraction to maintain the minimum divisional load (for a given number of progenitor compartments).

Interestingly, although optimal tissues have a constant self-renewal across progenitor cells, the division rates of the progenitor cells do not affect the divisional load and are thus unconstrained by our optimization procedure. Briefly, the progenitor compartment size $N_{i}^{*}$ is inversely proportional to the division rate in that compartment (equation (2.3)), so the total differentiation flux out of that compartment [$(1-\alpha_{i})\beta_{i}N_{i}^{*}$] is independent of *β*_*i*_. Therefore, the relationship between compartment sizes in these optimized tissues are unconstrained as well: i.e. it is not necessary to have a monotonically increasing compartment size for the tissue to minimize divisional load. In optimized tissue structures (binary division trees), we do have a doubling of the flux of cells between consecutive progenitor compartments. By assuming a constant division rate across progenitor compartments, we arrive at the doubling of subsequent progenitor compartment sizes in these optimized tissues.

Our conclusions are in agreement with those of Derényi & Szöllősi [6], despite different optimization procedures: we both find that hierarchically organized tissues can be tuned to minimize divisional loads, and therefore limit the occurrence of somatic mutations during homeostasis. Beyond Derényi & Szöllősi [6], we show that the constant ratio of differentiation outputs between subsequent compartments can be generalized further: we find that the ratio of differentiation output to net loss per compartment is conserved across all compartments, which in the absence of asymmetric divisions means that self-renewal probability is constant across all compartments. Furthermore, we explore the impacts of varying the optimization timescale, lifetime, SC numbers and SC quiescence.

Although the number of divisions itself is an important measure for damage accumulation per cell, it is not the full story in terms of cancer prevention. Intuitively, it is not clear if the total number of divisions per TDC, or only the number of divisions in the SC compartment, is important for cancer initiation and incidence. Tomasetti & Vogelstein have shown that across tissues the dependence of cancer incidence on the number of SC divisions is sub-linear, such that an increase in SC divisions does not lead to a proportional increase in cancer risk [24,25]. Therefore, other factors are at play, which could be extrinsic (e.g. smoking), or related to tissue architecture. Nowak *et al.* [8] formalized the concepts put forward by Cairns [5] to show that preventing self-renewal in all cells downstream of the SC minimizes the rate that mutations fixate in the population. This simple so-called ‘linear model’ of somatic evolution corresponds to our optimized binary division tree. Later, Pepper *et al.* [9] extended the serial differentiation model to include cell compartments (as in our model). They showed that non-self-renewing tissue structures can suppress cell level selection and somatic evolution, as serial differentiation makes it possible to segregate proliferative activity and population self-renewal into different cell compartments, such that no compartment possesses all the attributes necessary for somatic evolution. Any progenitor self-renewal, including asymmetric divisions, would allow rapid somatic evolution because a mutant clone can sweep to fixation within a population of such cells. Therefore, the tissue structures which we find limit the divisional load also limit the rate of somatic evolution.

Recently, Alvarado *et al.* [14] used an alternative ‘top-down’ modelling approach in which cells that are lost from the TDC compartment are replaced by pulling from the previous compartment (rather than SC division pushing cells into the next compartment). They showed that by increasing progenitor self-renewal it becomes unlikely that mutations are acquired in the more primitive compartments, but mutations that do occur take longer to be flushed out of the tissue. Overall, large values of progenitor self-renewal result in a smaller number of single-hit mutants across all compartments. However, from the point of view of two-hit mutant generation (e.g. the inactivation of a tumour suppressor gene), less progenitor self-renewal is advantageous as the lifetime of the transient cells is shorter, and they therefore have less time to accumulate two mutations. This optimal architecture for delaying two-hit mutations corresponds to the binary division tree which limits divisional load. Interestingly, Alvarado *et al.* [14] found that the arrangement of progenitor compartment sizes influences the total number of mutants and the rate at which two-hit mutants are generated. This is seemingly in contrast with our observation that progenitor compartment sizes are essentially a free variable and unconstrained by minimizing the divisional load. This discrepancy can be explained by the fact that the cell division rates in Alvarado *et al.* [14] are determined by a feedback loop and are intrinsically coupled to the self-renewal probabilities.

In our study, we have quantified only the mean divisional load per TDC. Böttcher *et al.* [15] showed that, based on telomere length distributions, there is significant heterogeneity among divisional loads. In their analysis, Böttcher *et al.* [15] showed that not only the mean, but also the variance, increases with increasing progenitor self-renewal, leading to few cells with very high divisional load. This again suggests that the non-self-renewing tissue would be the optimum for minimizing the probability of a cell evolving an oncogenic phenotype through mutation accumulation.

Our analysis relies on assumptions about the system being in steady state and parameter values being constant (for example, constant HSC activation and deactivation throughout life). This, however, is not representative of early development when SCs undergo significant expansion and tissue compartments are populated. An expansion phase would require an initially increased SC self-renewal rate to allow the SCs to reach the homeostatic level and to populate the progenitor and TDC compartments.

With this study, we have shown that limiting progenitor self-renewal in hierarchical tissues results in the lowest divisional load during homeostatic tissue maintenance. This optimized tissue also satisfies some other somatic evolution constraints put forward by multiple authors. Going forward, we would like to bring all of these aspects together to achieve an overall understanding of the selection pressures that act on and shape tissue organization.

## Data accessibility

Code is publicly available at https://github.com/ashcroftp/optimising-divisions and archived at https://doi.org/10.5281/zenodo.5554123.
